# Supplementary material for: A High-Throughput Screening of a Natural Products Library for Mitochondria Modulators
Source: Biomolecules. 2024 Apr 4;14(4):440. doi: 10.3390/biom14040440 (PMC11048375; doi:10.3390/biom14040440)
Supplement: Supplementary file 1 [file biomolecules-14-00440-s001.zip › biomolecules-2904557-supplementary.pdf]

Supplementary Material

A High-Throughput Screening of a Natural Products Library for Mitochondria Modulators

Emmanuel Makinde [1], Linlin Ma [1,2], George D. Mellick [1,2] and Yunjiang Feng [1,2,*]

[1] Griffith Institute for Drug Discovery, Griffith University, Brisbane, QLD 4111, Australia
[2] School of Environment and Science, Griffith University, Brisbane QLD 4111, Australia
[*] Correspondence: y.feng@griffith.edu.au

Table of contents.

Figure S1. HPLC Chromatogram of *Ternstroemia sp.* 2
Figure S2. HPLC Chromatogram of *Alnus sp.* 2
Figure S3. HPLC Chromatogram of *Balanops sp.* 2
Figure S4. HPLC Chromatogram of *Anredera sp.* 2
Figure S5. HPLC Chromatogram of *Cestrum sp.* 3
Figure S6. HPLC Chromatogram of *Ilex sp.* 3
Figure S7. HPLC Chromatogram of *Dendrilla sp.* 3
Figure S8. HPLC Chromatogram of *Balanophora sp.* 3
Figure S9. HPLC Chromatogram of *Aaptos sp.* 4
Figure S10. HPLC Chromatogram of *Rhaphoxya sp.* 4
Figure S11. HPLC Chromatogram of *Fucraea sp.* 4
Figure S12. MTT assay of fractions at 100, 50 and 25 µg/ml 5
Figure S12 (contd.). MTT assay of fractions at 100, 50 and 25 µg/ml continued. 6
Figure S13. Base peak ion chromatogram and full mass spectra of *Ternstroemia sp.*, fraction 4. 7
Figure S14. Base peak ion chromatogram and full mass spectra of *Alnus sp.*, fraction 4 8
Figure S15. Base peak ion chromatogram and full mass spectra of *Anredera sp.*, fraction 1. 9
Figure S16. Base peak ion chromatogram and full mass spectra of *Cestrum sp.*, fraction 1. 10
Figure S17. Base peak ion chromatogram and full mass spectra of *Dendrilla sp.*, fraction 3. 11
Figure S18. Base peak ion chromatogram and full mass spectra of *Balanophora sp.*, fraction 2 12
Figure S19. Base peak ion chromatogram and full mass spectra of *Aaptos sp.*, fraction 6 13
Figure S20. Base peak ion chromatogram and full mass spectra of *Rhaphoxya sp.*, fraction 6 14

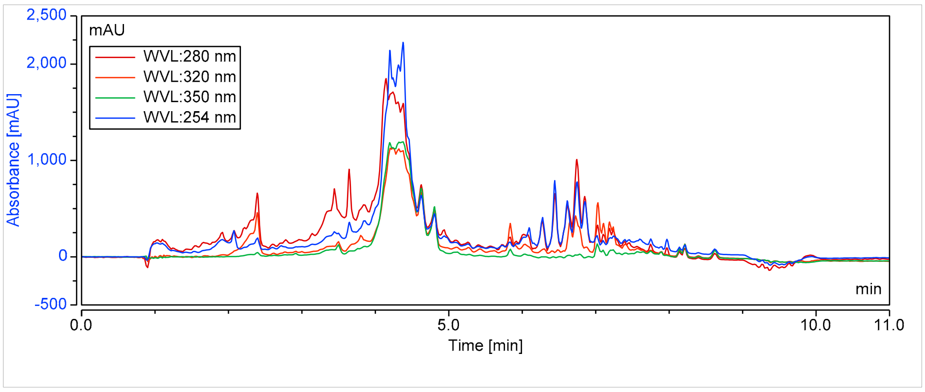

Figure S1. HPLC Chromatogram of *Ternstroemia sp.*

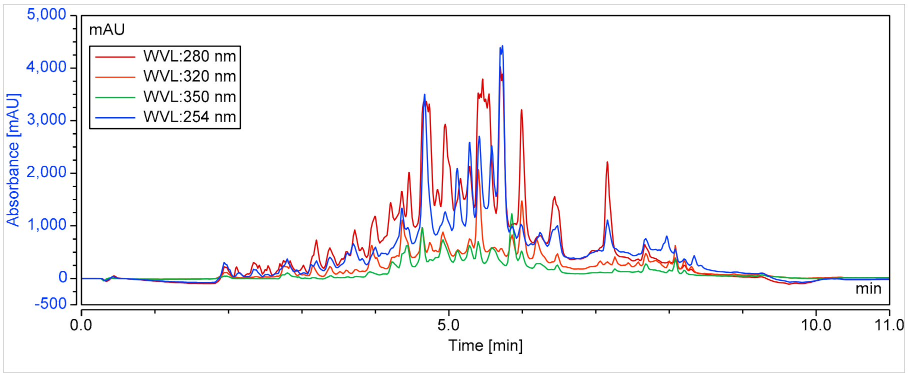

Figure S2. HPLC Chromatogram of *Alnus sp.*

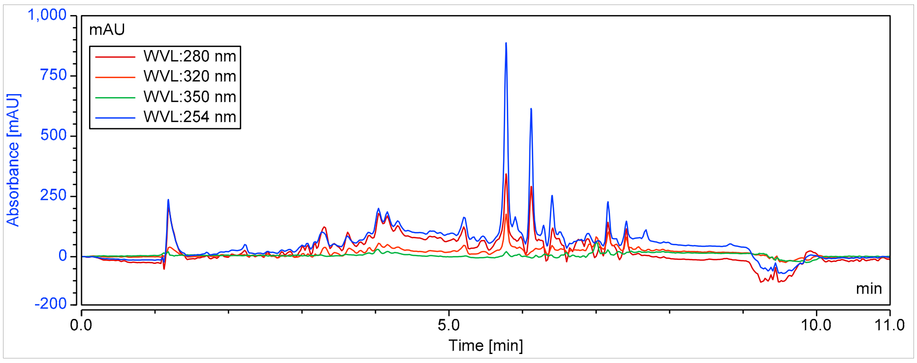

Figure S3. HPLC Chromatogram of *Balanops sp.*

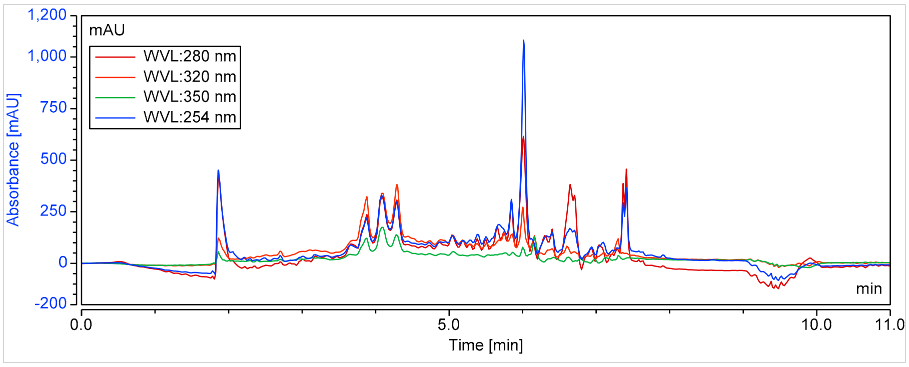

Figure S4. HPLC Chromatogram of *Anredera sp.*

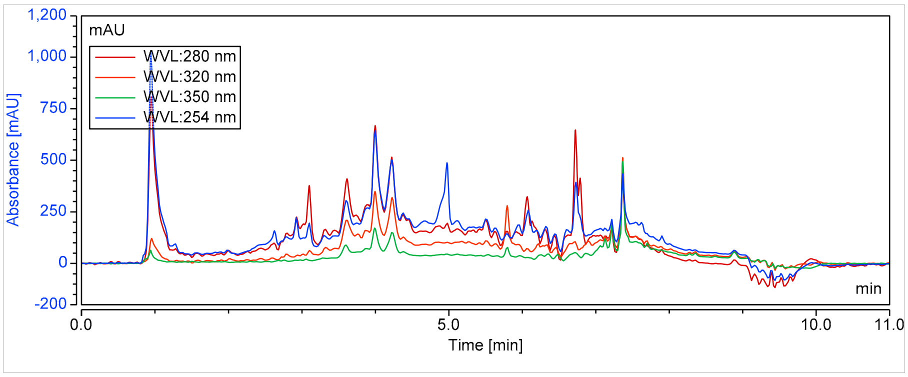

Figure S5. HPLC Chromatogram of *Cestrum sp.*

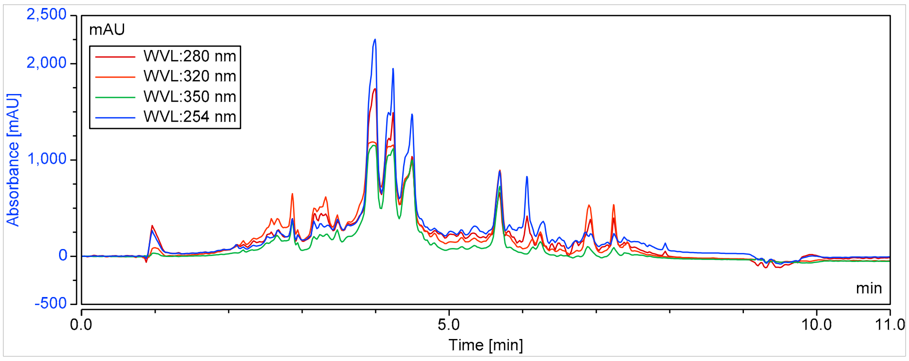

Figure S6. HPLC Chromatogram of *Ilex sp.*

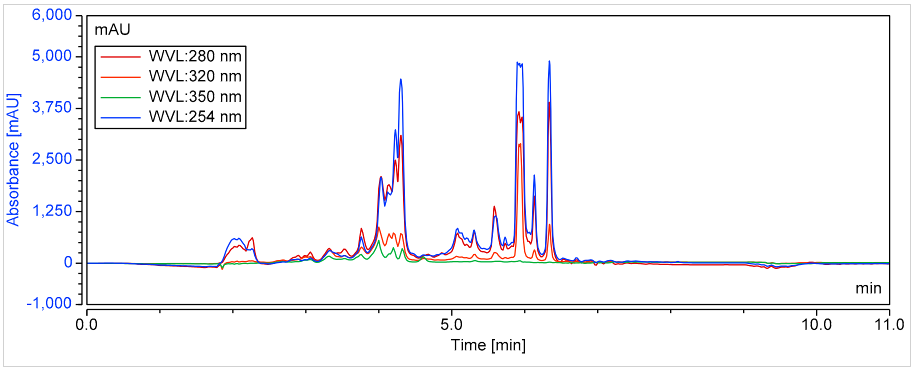

Figure S7. HPLC Chromatogram of *Dendrilla sp.*

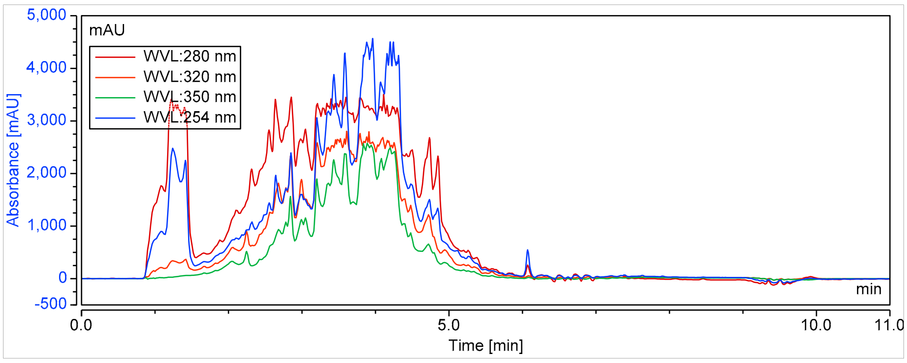

Figure S8. HPLC Chromatogram of *Balanophora sp.*

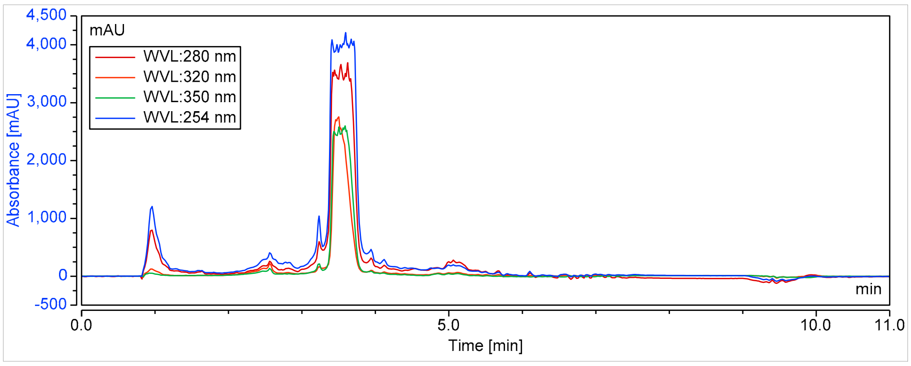

Figure S9. HPLC Chromatogram of *Aaptos sp.*

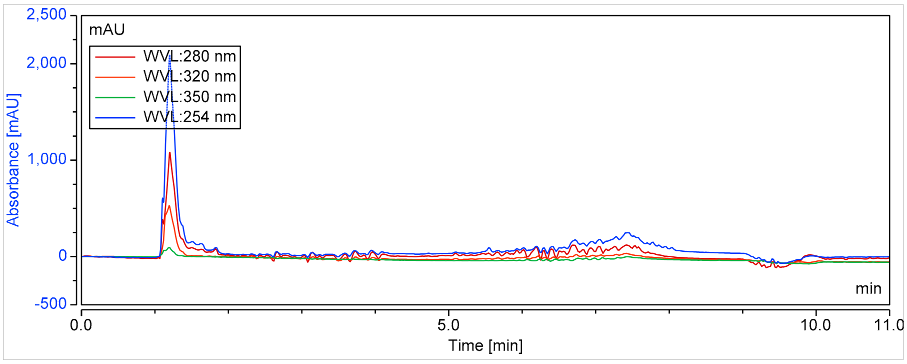

Figure S10. HPLC Chromatogram of *Rhaphoxya sp.*

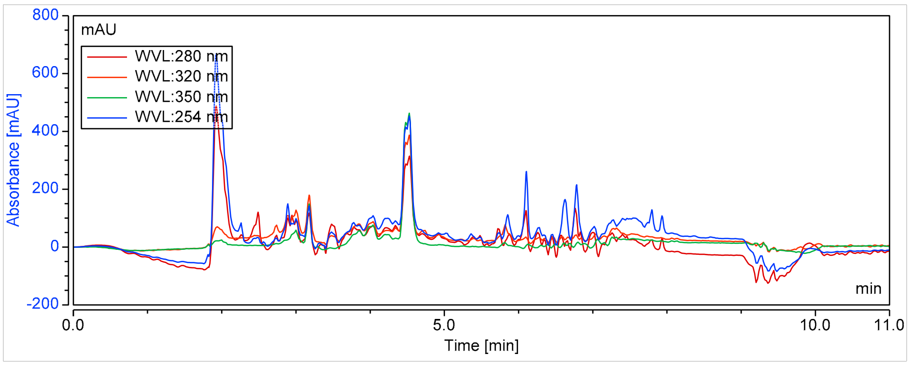

Figure S11. HPLC Chromatogram of *Fucraea sp.*

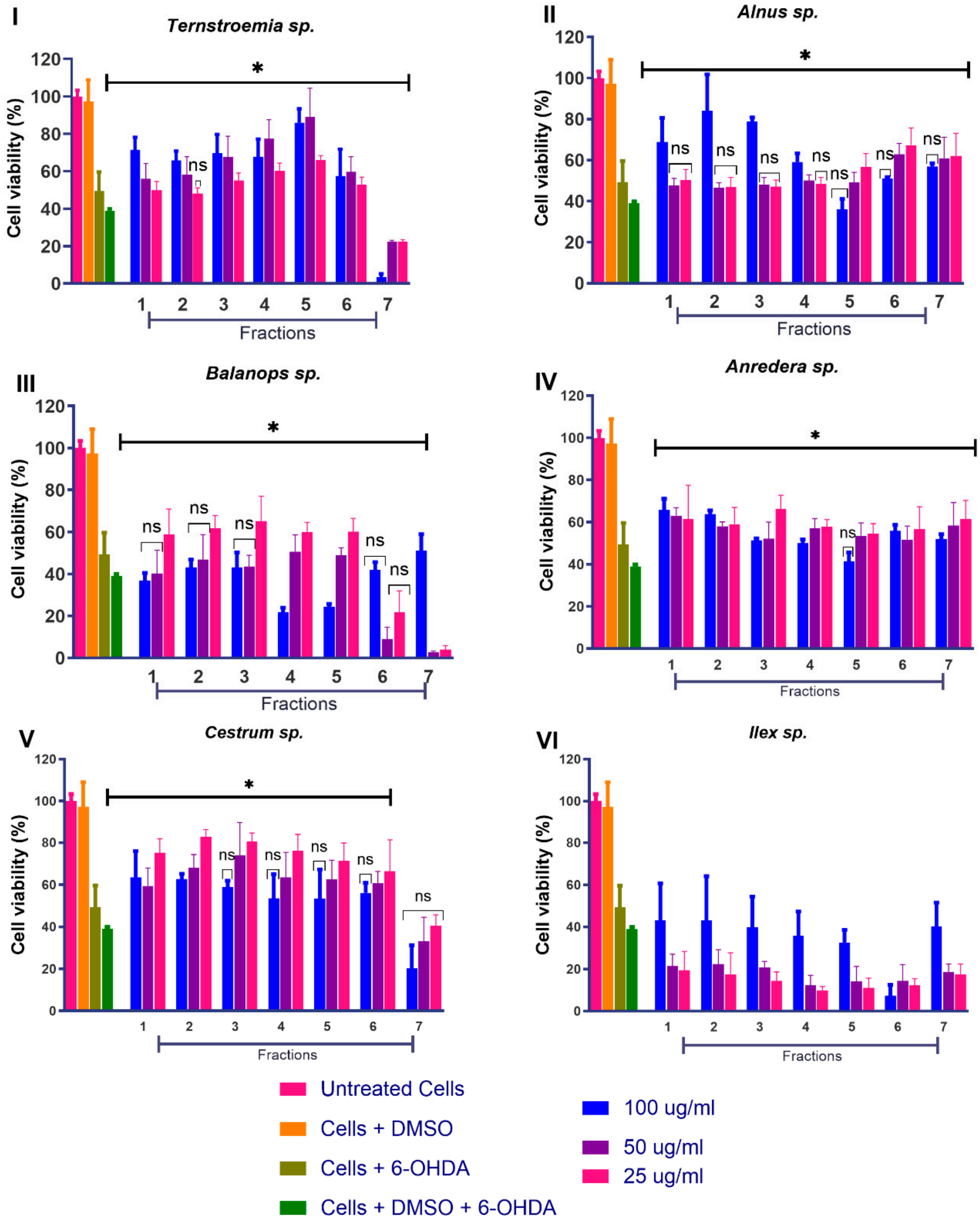

Figure S12. MTT assay of fractions at 100, 50 and 25 μg/ml

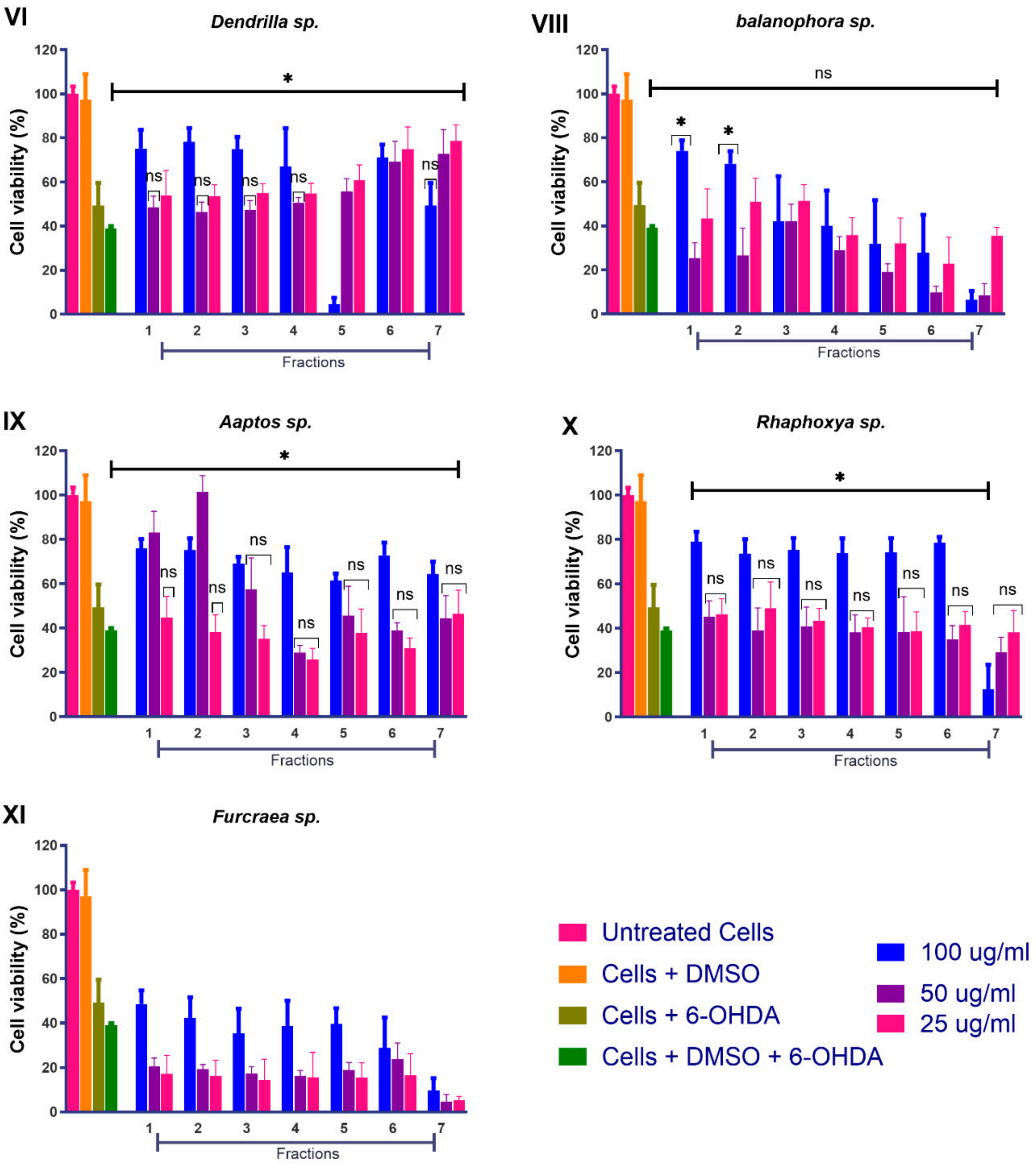

Figure S12 (contd). MTT assay of fractions at 100, 50 and 25 µg/ml.

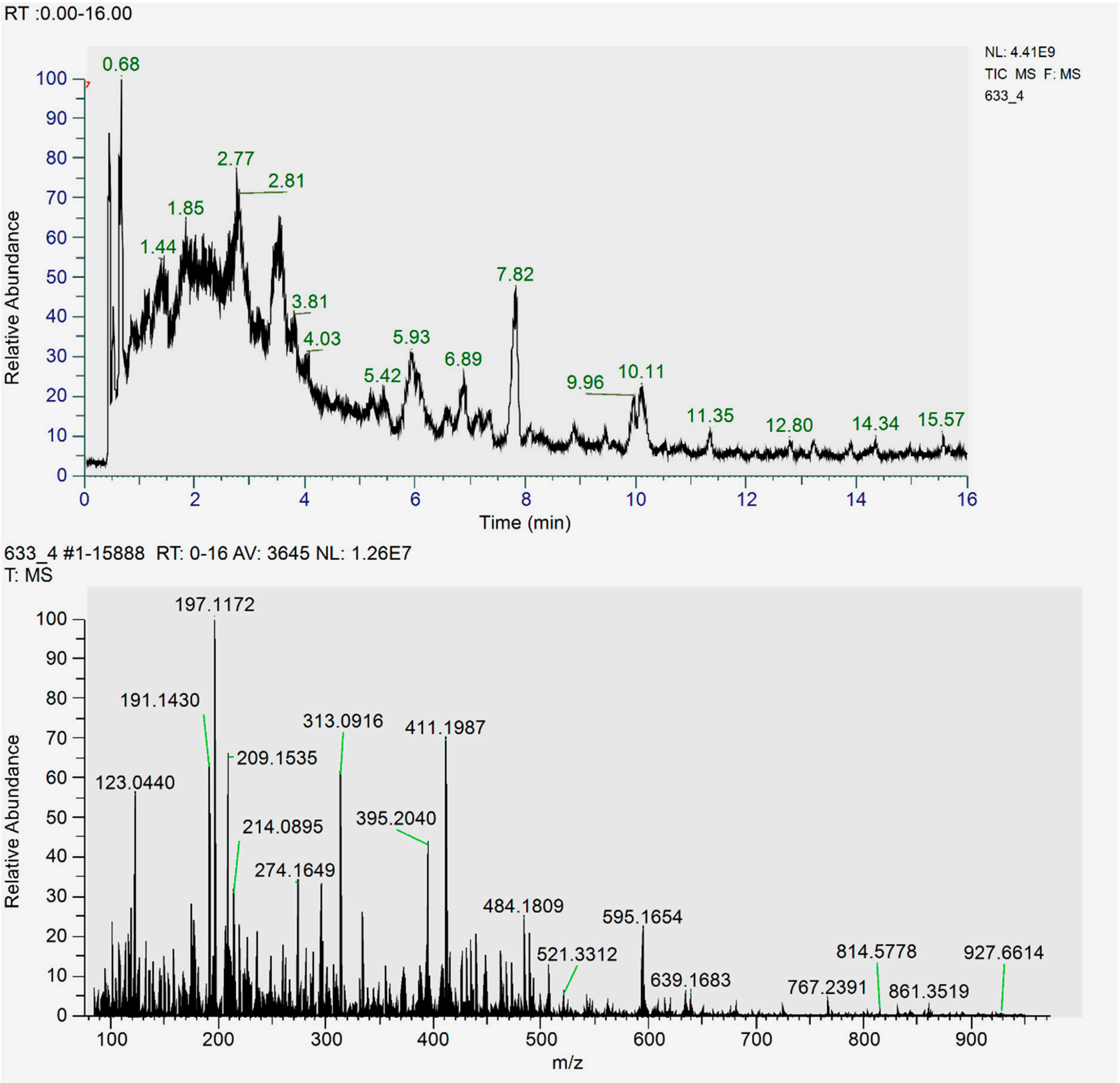

Figure S13. Base peak ion chromatogram and full mass spectra of *Ternstroemia sp.*, fraction 4.

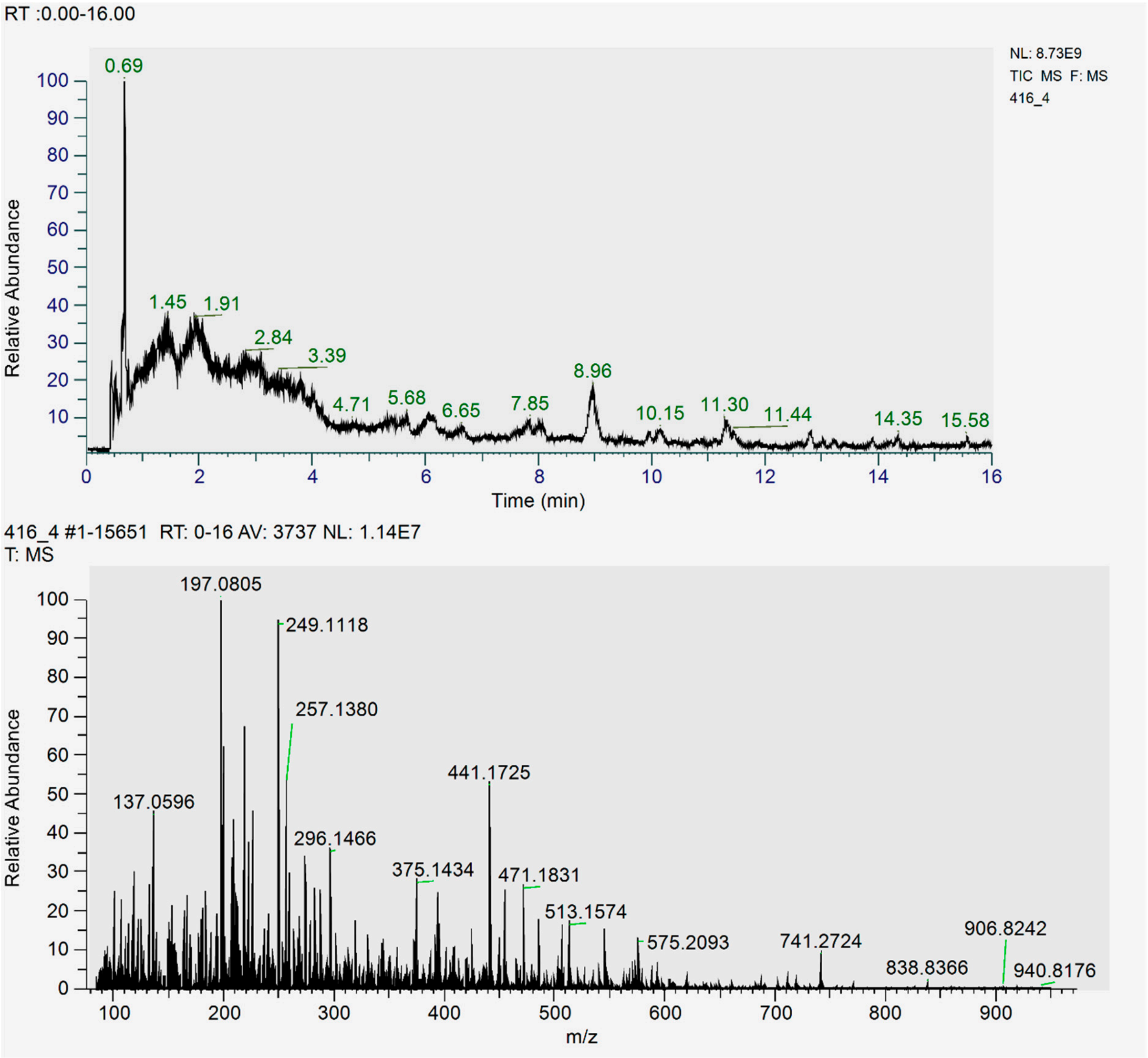

Figure S14. Base peak ion chromatogram and full mass spectra of *Alnus sp*., fraction 4.

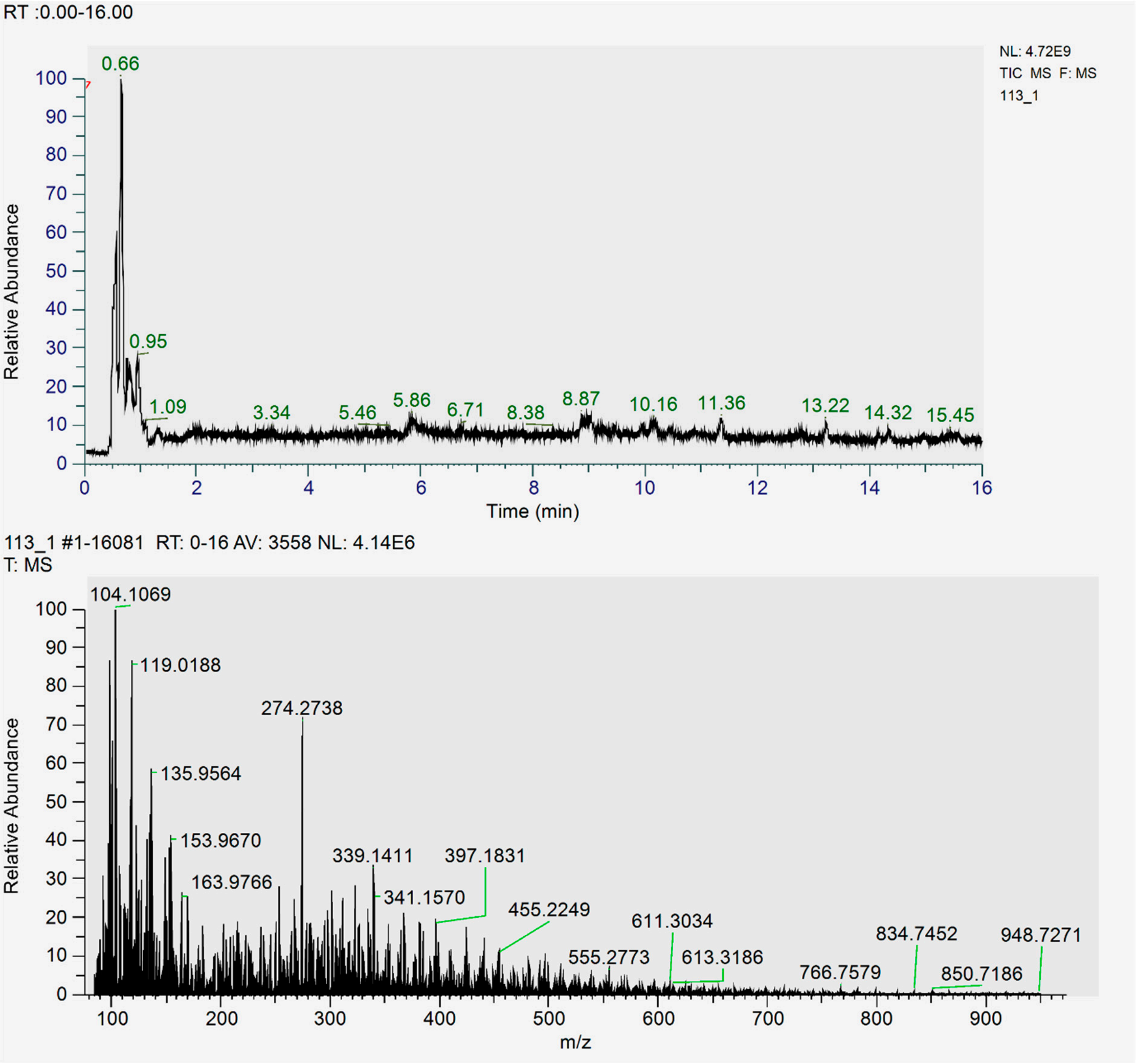

Figure S15. Base peak ion chromatogram and full mass spectra of *Anredera sp*., fraction 1.

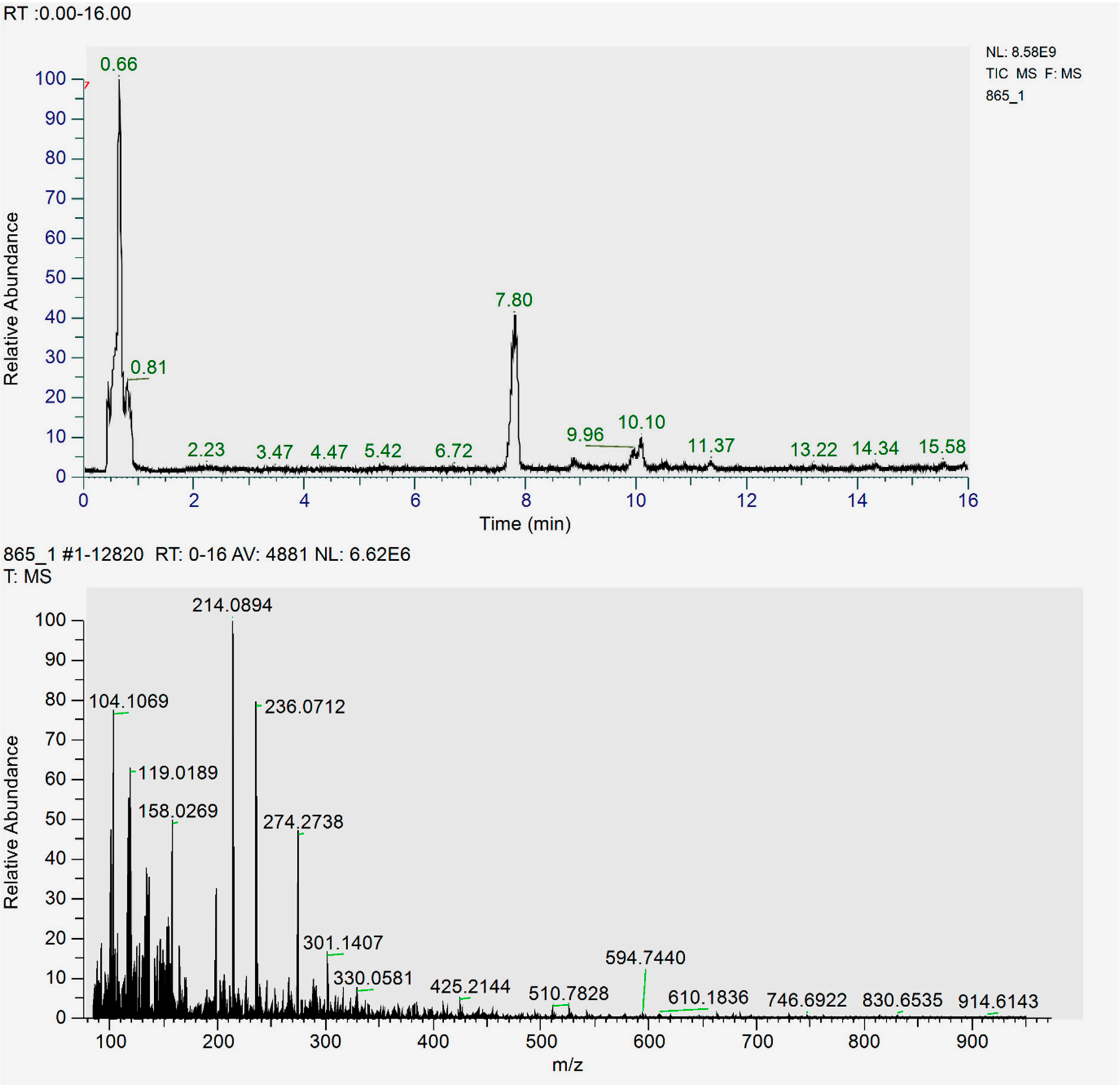

Figure S16. Base peak ion chromatogram and full mass spectra of *Cestrum sp*., fraction 1.

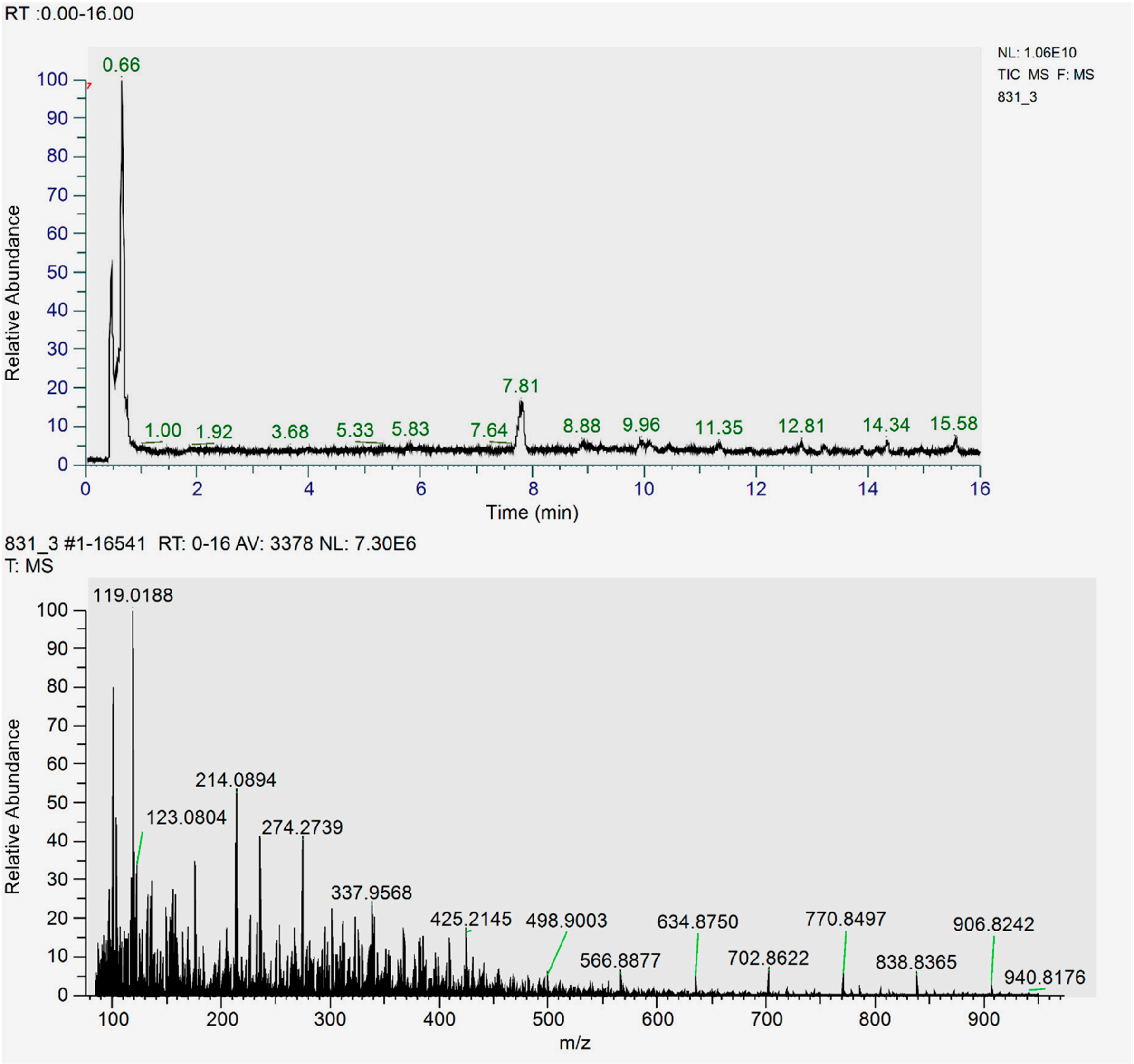

Figure S17. Base peak ion chromatogram and full mass spectra of *Dendrilla sp*., fraction 3.

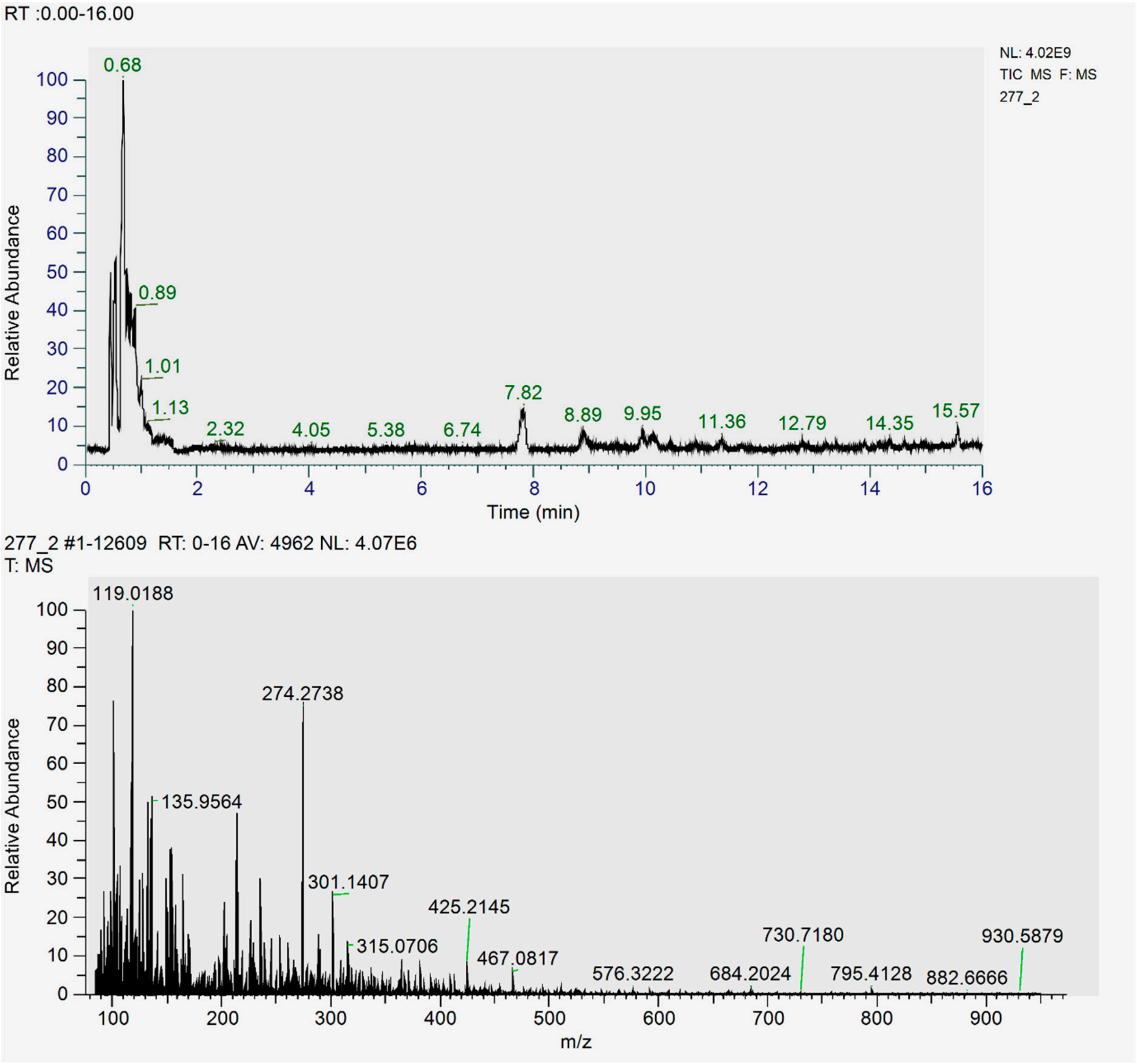

Figure S18. Base peak ion chromatogram and full mass spectra of *Balanophora sp*., fraction 2.

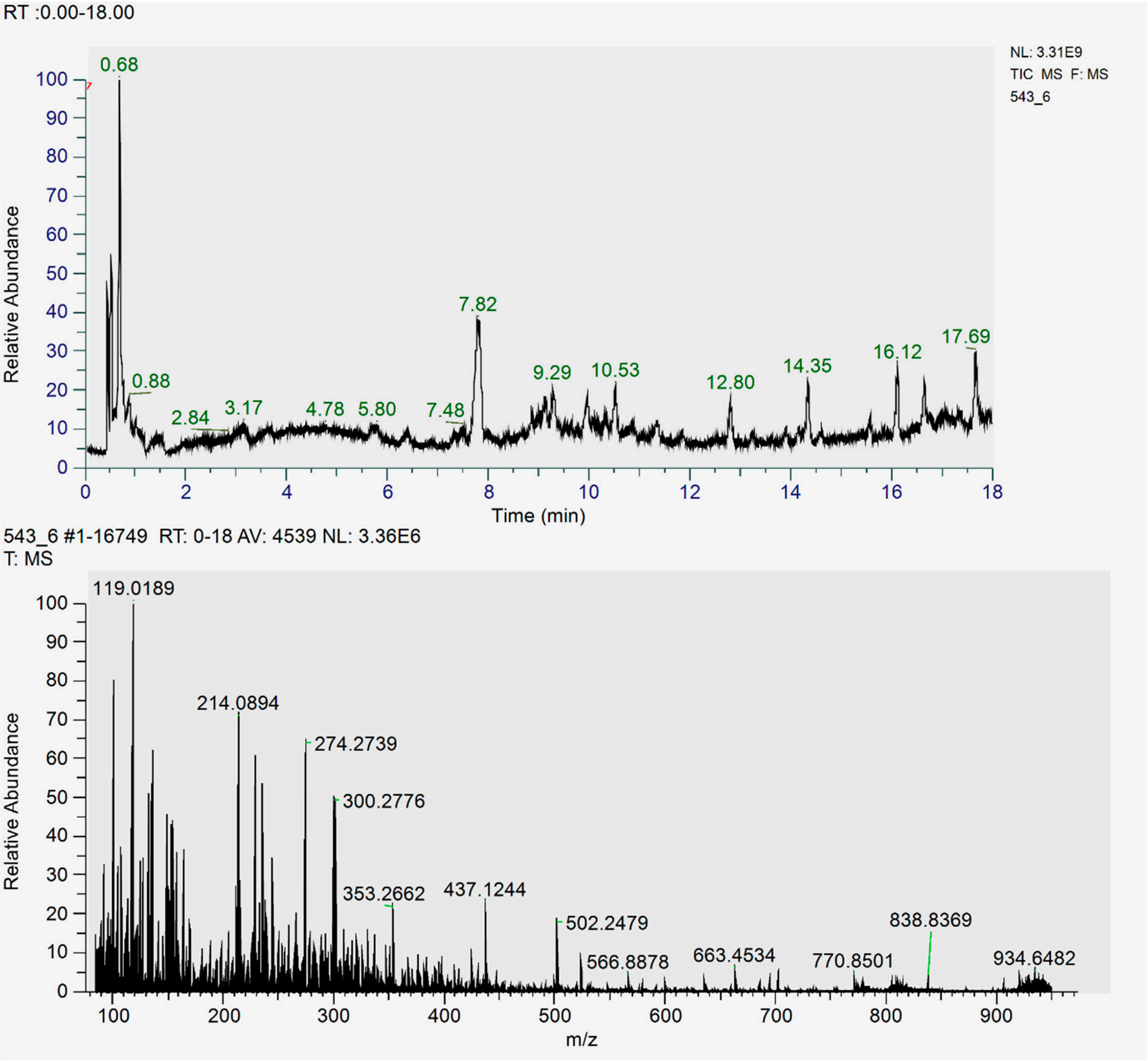

Figure S19. Base peak ion chromatogram and full mass spectra of *Aaptos sp*., fraction 6.

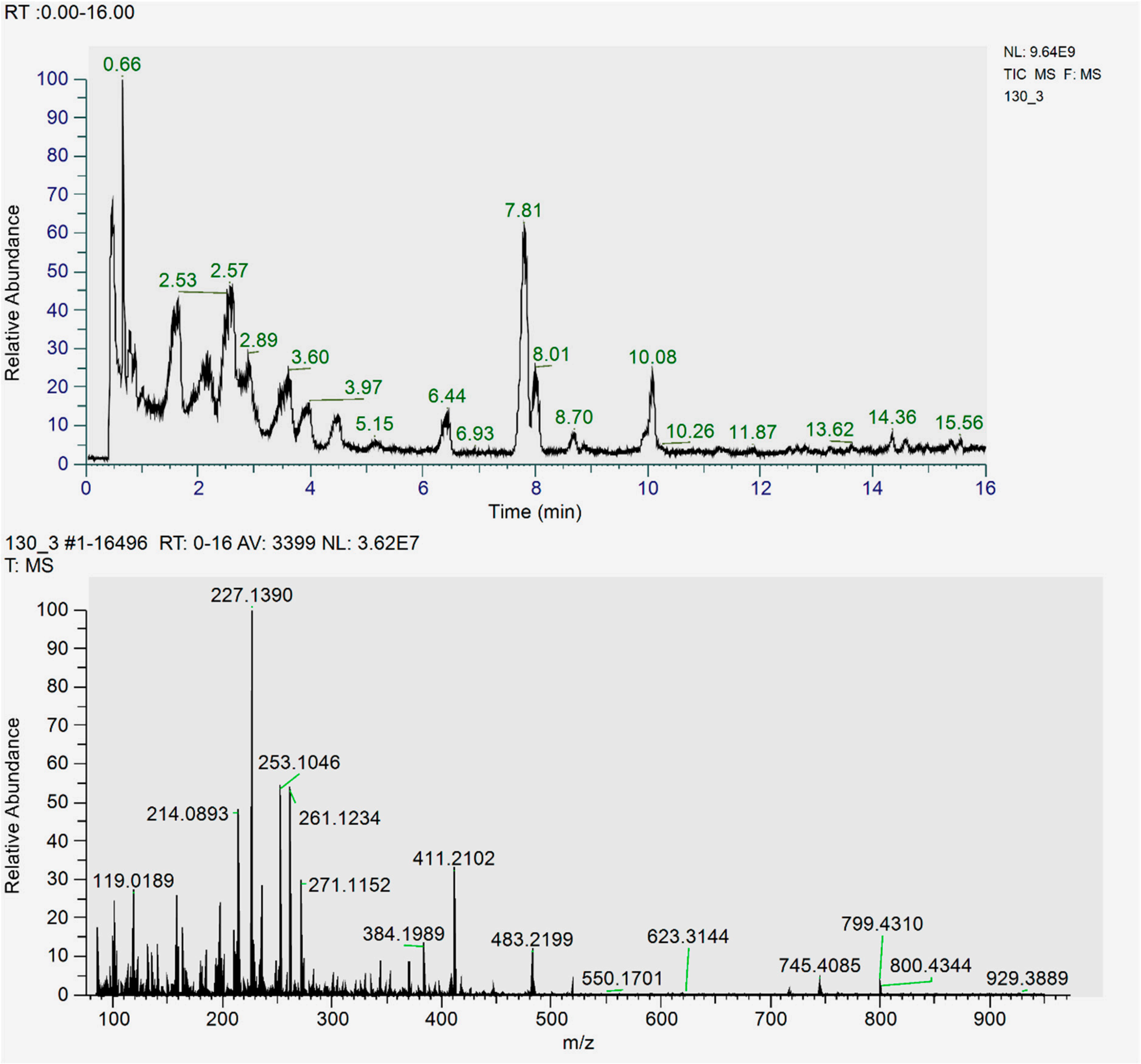

Figure S20. Base peak ion chromatogram and full mass spectra of *Rhaphoxya sp.,* fraction 6.
